# Characterization of biofilm-like structures formed by *Pseudomonas aeruginosa* in a synthetic mucus medium

**DOI:** 10.1186/1471-2180-12-181

**Published:** 2012-08-18

**Authors:** Cecily L Haley, Jane A Colmer-Hamood, Abdul N Hamood

**Affiliations:** 1 Department of Immunology and Molecular Microbiology, Texas Tech University Health Sciences Center, School of Medicine, Lubbock, TX, USA

## Abstract

**Background:**

The accumulation of thick stagnant mucus provides a suitable environment for the growth of *Pseudomonas aeruginosa* and *Staphylococcus aureus* within the lung alveoli of cystic fibrosis (CF) patients. These infections cause significant lung damage, leading to respiratory failure and death. In an artificial mucin containing medium ASM+, *P. aeruginosa* forms structures that resemble typical biofilms but are not attached to any surface. We refer to these structures as biofilm like structures (BLS). Using ASM+ in a static microtiter plate culture system, we examined the roles of mucin, extracellular DNA, environmental oxygen (EO_2_), and quorum sensing (QS) in the development of biofilm-like structures (BLS) by *P. aeruginosa*; and the effect of EO_2_ and *P. aeruginosa* on *S. aureus* BLS.

**Results:**

Under 20% EO_2_, *P. aeruginosa* strain PAO1 produced BLS that resemble typical biofilms but are confined to the ASM+ and not attached to the surface. Levels of mucin and extracellular DNA within the ASM+ were optimized to produce robust well developed BLS. At 10% EO_2_, PAO1 produced thicker, more developed BLS, while under 0% EO_2_, BLS production was diminished. In contrast, the *S. aureus* strain AH133 produced well-developed BLS only under 20% EO_2_. In PAO1, loss of the QS system genes *rhlI* and *rhlR* affected the formation of BLS in ASM+ in terms of both structure and architecture. Whether co-inoculated into ASM+ with AH133, or added to established AH133 BLS, PAO1 eliminated AH133 within 48–56 h.

**Conclusions:**

The thick, viscous ASM+, which contains mucin and extracellular DNA levels similar to those found in the CF lung, supports the formation of biofilm-like structures similar to the aggregates described within CF airways. Alterations in environmental conditions or in the QS genes of *P. aeruginosa*, as occurs naturally during the progression of CF lung infection, affect the architecture and quantitative structural features of these BLS. Thus, ASM+ provides an *in vitro* medium in which the effect of changing levels of substances produced by the host and the bacteria can be analyzed to determine the effect on such structures and on the susceptibility of the bacteria within the BLS to various treatments.

## Background

Cystic fibrosis (CF), an inherited disorder caused by mutations in the gene that encodes the cystic fibrosis transmembrane conductance regulator, affects approximately 30,000 Americans, primarily those of Northern European origin [1,2]. These mutations cause a deficiency in chloride secretion with ensuing accumulation of thick, stagnant mucus within the lung alveoli of the patients [1-4]. Nutrients in the thick mucus facilitate the colonization of various bacterial pathogens, including *Pseudomonas aeruginosa, Staphylococcus aureus,* and *Haemophilus influenzae*[3,5]. Colonization by these pathogens elicits a strong host inflammatory response which leads to destruction of the lung tissue and, ultimately, death from respiratory failure [1,6,7]. *P. aeruginosa* is one of the significant pathogens in chronic lung infections of CF patients [1,8].

Among the different factors that contribute to the virulence of *P. aeruginosa* is its ability to form a biofilm, a community within which bacteria are attached to a substratum or to each other [9]. Within the biofilm, the bacteria are surrounded by extracellular polymeric substance (EPS), which protects them from the effects of the host immune system and from diverse antibiotics [10-12]. Biofilm development occurs in stages that require specific bacterial factors at each stage. For example, during the initial (attachment) stage of biofilm formation, bacteria depend on both the flagellum-mediated swimming motility and the pili-mediated twitching motility [13]. A number of *P. aeruginosa* infections are associated with biofilm formation, including chronic otitis media, heart valve endocarditis, and chronic lung infections in CF patients [9,14].

Previously, studies have described synthetic mucin-containing artificial sputum media (ASM) that mimics the thick mucus within the lung of CF patients [15,16]. When grown in ASM, *P. aeruginosa* formed in tight microcolonies suspended within the medium rather than attached to the surface or free swimming as in standard broth media [15,16]. Mucin is the main component of secreted mucus, which also contains a large number of plasma and non-plasma proteins, carbohydrates, amino acids, nucleic acids, lipids, and electrolytes [17,18]. It has been shown that mucin-*P. aeruginosa* interactions promote biofilm formation in the continuous culture flow-through system [19].

In this study, we utilized a static microtiter plate culture system to investigate the effect of different conditions on the development of *P. aeruginosa* biofilms in mucus medium. Within the medium, *P. aeruginosa* strain PAO1 formed structures that are biofilm-like, but are not attached to the surface. The amount of mucin and extracellular DNA in the medium, as well as the level of environmental oxygen (EO_2_), are critical for the development of these biofilm-like structures (BLS). Additionally, one of the *P. aeruginosa* quorum sensing systems, *rhl*, affects formation of the BLS. Furthermore, as it develops its BLS, *P. aeruginosa* eliminates already established *S. aureus* BLS by a bactericidal mechanism.

## Results

**Figure 1:**
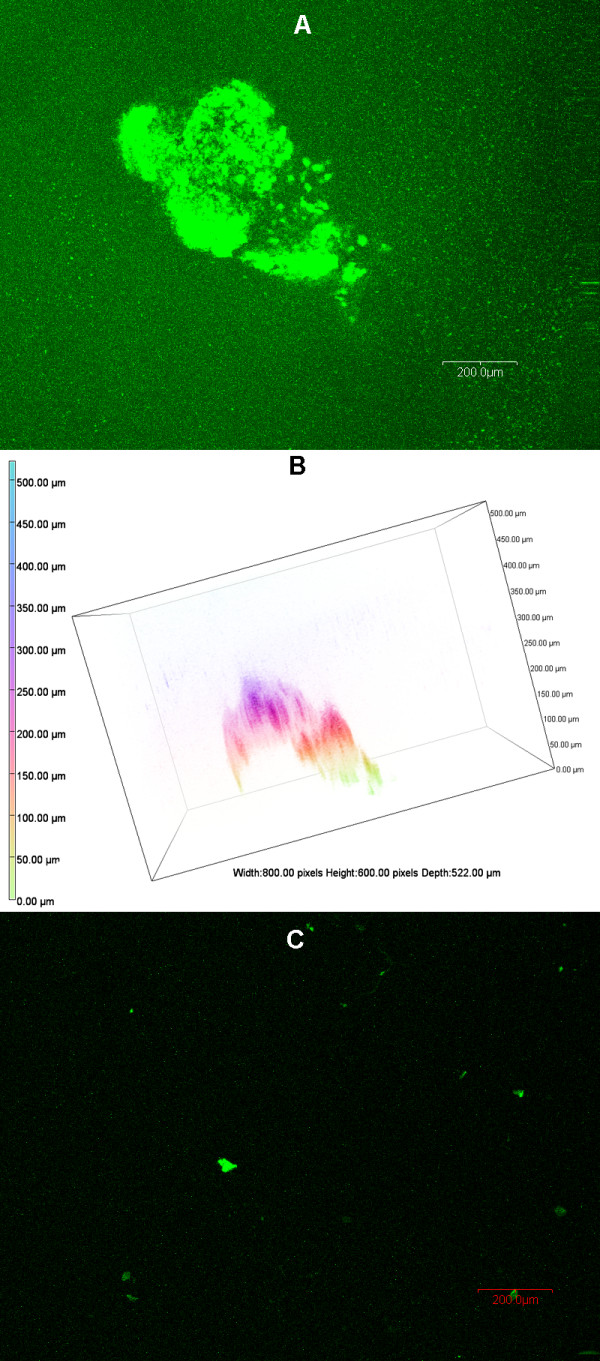
***P. aeruginosa *PAO1 forms BLS within the ASM+.** After 48 h of growth at 37°C under 20% EO_2_/static conditions, PAO1/pMRP9-1 developed BLS that were confined to the ASM+ and not attached to the surface of the microtiter plate. The composition of the ASM+ and the bacterial inoculation are described in Methods. The gelatinous mass containing the BLS was visualized *in situ* by CLSM. (**A**) CLSM micrograph of the PAO1/pMRP9-1 BLS; magnification, 10X; bar, 200.00 nm. (**B**) 3-D image analysis revealing the architecture of the BLS shown in (A); box, 800.00 pixels (px) W x 600 px H; bar, 100 px. (**C**) CLSM micrograph of the well bottom after the removal of the gelatinous mass showing no attached bacteria or biofilm (the scattered fluorescence observed is due to autofluorescing debris).

Previous studies described a synthetic medium, ASM, which closely mimics the sputum of CF patients [15,16]. When grown in ASM, PAO1 formed clusters, or microcolonies, that are attached to the components of the ASM but not the abiotic surface [16]. In this study, we analyzed the influence of different conditions on the formation of these unique structures. We then examined the growth of the *P. aeruginosa* strain PAO1/pMRP9-1 in the static microtiter plate culture system using ASM+. First, we eliminated the possibility that the addition of antibiotics (either carbenicillin or erythromycin) to ASM+ to maintain the GFP plasmid had an adverse effect on either the growth of the tested strains or BLS development by these strains (data not shown). Inoculated plates were incubated at 37°C under 20% EO_2_. *In situ* CLSM of the gelatinous masses at 48 h revealed the formation of structures composed of numerous coalescing microcolonies that closely resemble mature well-developed PAO1 biofilms (Figure 1A, B). Quantitative analysis of the BLS using the COMSTAT program [20], supported these findings: a total biovolume of 6.52 ± 0.43 μm^3^/μm^2^ and a mean thickness of 11.57 ± 0.28 μm was seen at 48 h (Table 1). Unlike the development of PAO1 biofilms in other media, these unique suspended biofilm-like structures (BLS) are induced only within the gelatinous mass, as PAO1 did not form any biofilm on the surface of the microtiter well (Figure 1C). To further confirm these results, we grew PAO1 as described above but inserted a small plastic coverslip within the well of the microtiter plate. PAO1 again formed the unique BLS but failed to form any biofilm on the plastic surface (data not shown). In contrast, when we repeated the experiment with TSBDC, an iron-deficient medium in which *P. aeruginosa* grows planktonically and develops conventional biofilm, PAO1 formed a thick mature biofilm attached to the coverslip surface (data not shown).

**Table 1:** Effect of time and environmental variables on PAO1/pMRP9-1 BLS

**Variable**	**Image stacks (#)**^***a***^	**Total biovolume (μm**^**3**^**/μm**^**2**^**)**^***b***^	**Mean thickness (μm)**^***c***^	**Roughness coefficient**^***d***^	**Total surface area × 10**^**7**^**(μm**^**2**^**)**^***e***^	**Surface to volume ratio (μm**^**2**^**/μm**^**3**^**)**^***f***^
**Time (under 20%EO**_**2**_**)**						
48 h	10	6.52 ± 0.43	11.6 ± 0.28	0.53 ± 0.02	1.65 ± 0.24	1.54 ± 0.10
72 h	10	11.1 ± 0.40	15.5 ± 0.23	0.18 ± 0.02	2.15 ± 0.03	1.01 ± 0.04
6 d	10	18.2 ± 0.32	17.8 ± 0.06	0.02 ± 0.00	0.96 ± 0.12	0.28 ± 0.04
**Mucin concentration (3 d under 20%EO**_**2**_**)**						
1X	10	11.1 ± 0.40	15.5 ± 0.23	0.18 ± 0.02	2.15 ± 0.03	1.01 ± 0.04
0.5X	10	13.5 ± 0.24	17.0 ± 0.05	0.08 ± 0.00	2.44 ± .045	0.94 ± 0.03
2X	10	15.4 ± 0.35	17.3 ± 0.08	0.06 ± 0.00	1.97 ± .098	0.67 ± 0.05
**DNA concentration (3 d under 20%EO**_**2**_**)**						
1X	10	11.1 ± 0.40	15.5 ± 0.23	0.18 ± 0.02	2.15 ± 0.03	1.01 ± 0.04
0.5X	10	2.42 ± 0.54	4.37 ± 1.37	1.33 ± 0.20	0.76 ± .220	1.55 ± 0.15
1.5X	10	2.48 ± 0.22	5.52 ± 0.64	1.07 ± 0.07	0.96 ± .086	2.02 ± 0.01
**Oxygen concentration (EO**_**2**_**)**^***g***^						
20%	10	11.1 ± 0.40	15.5 ± 0.23	0.18 ± 0.02	2.15 ± 0.03	1.01 ± 0.04
10%	10	19.4 ± 0.28	17.9 ± 0.04	0.01 ± 0.00	0.46 ± 0.12	0.13 ± 0.03
0%	10	0.28 ± 0.19	0.41 ± 0.27	1.94 ± 0.04	0.07 ± 0.06	1.75 ± 0.30

^*a*^ Each experiment was done in duplicate. Two 10-image stacks were obtained from random positions within the BLs. A total of 40-image stacks were analyzed were analyzed using the COMSTAT program [20]. Values represent the mean ± SEM.

^*b*^Estimates the biomass of the BLS.

^*c*^Measures spatial size of the BLS.

^*d*^Assessment of the variation in the thickness of the BLS, or BLS heterogeneity.

^*e*^Total of the area occupied in each image stack.

^*f*^Estimates the portion of the biofilm exposed to nutrients; biovolume divided by the surface area of the substratum.

^*g*^20%, aerobic; 10%, microaerobic; 0%, anaerobic; cultures were grown for 3 d, except 0% EO_2_ for 6d.

### Extended incubation time enhances the formation of the BLS

**Figure 2:**
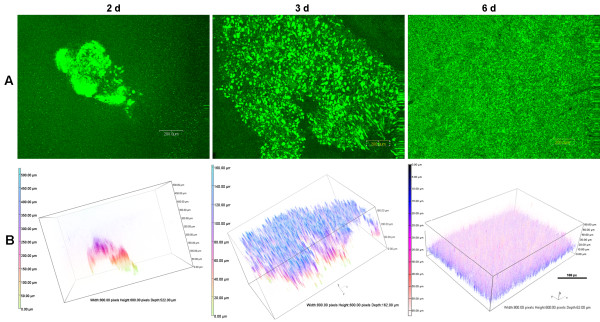
**PAO1 BLS vary structurally over time.** Bacterial inoculation and incubation for the development of BLS were done as described in Figure 1, except incubation was continued for 6 d without changing the medium. (**A**) CLSM micrographs of BLS at 2, 3, and 6 d post-inoculation; magnification, 10X; bars, 200.00 nm. (**B**) The 3-D architecture of the BLS shown in (A). Boxes, 800.00 px W x 600.00 px H; bars, 100 px.

**Figure 3:**
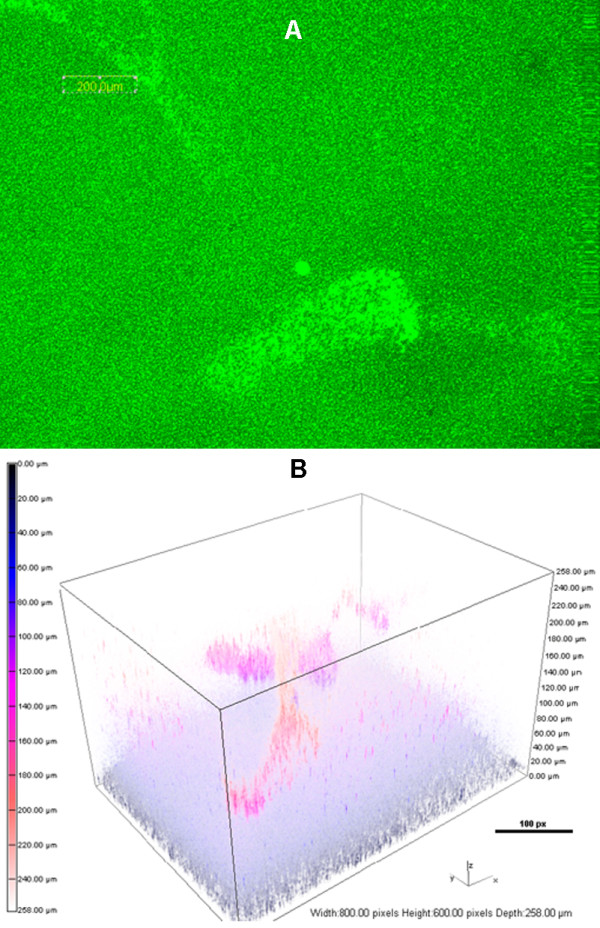
**Extending incubation to 16 d enhances the formation of PAO1 BLS.** Bacterial inoculation and incubation for the development of BLS were done as described in Figure 1, except fresh ASM+ was added to the wells at 4-d intervals to replace lost volume. (**A**) CLSM micrographs of BLS at 16 d post-inoculation; magnification, 10X; bar, 200.00 nm. (**B**) The 3-D architecture of the BLS shown in (A). Boxes, 800.00 px W x 600.00 px H; bar, 100 px.

One condition that may influence the development of the BLS in the ASM+ is length of incubation. Since the growth of PAO1 in ASM+ appears similar to the macrocolonies reported within the lungs of CF patients with chronic *P. aeruginosa* infection [21], we inoculated ASM+ with PAO1/pMRP9-1 as described above and incubated the cultures in 20% EO_2_ at 37°C for up to 16 d. From days 2 to 6, the BLS gradually developed to resemble a complete, mature and well developed biofilm (Figure 2A). Three-dimensional (3-D) images constructed from the CLSM scans clearly show the gradual increase in the size and the thickness of the BLS (Figure 2B). Structural analysis revealed that between 2–3 and 2–6 days, the BLS significantly increased in total biovolume and mean thickness (Tables 1 and 2). In contrast, portions of the BLS that are exposed to nutrients (the surface to biovolume ratio) and roughness coefficient values were significantly reduced (Tables 1 and 2). The total surface area was significantly (P < 0.0001) decreased between 2–6 days only (Table 1). For the 16-d growth experiments, we maintained the growth of the PAO1 BLS by adding fresh ASM+ to the media remaining in the wells to maintain the original volume every 4 d to replace volume lost to evaporation. At 16 d, PAO1 BLS appears to be greater than at any time during the course of the experiment (Figure 3). Due to enhanced growth by the replacement of the medium, new microcolonies appear to have developed atop the underlying thick growth (Figure 3). Alternatively, these microcolonies may represent detached segments of the well developed biofilm (Figure 3). Such detachment may occur mechanically and would not represent the well known bacterial dispersion phenomenon. In bacterial dispersion, individual planktonic cells and not biofilm segments are released from the mature biofilm [14]. No biofilm attached to the surface of the well of the microtiter plate at any time point throughout the experiment (data not shown). These results suggest that dynamic changes within occur PAO1 BLS during growth in ASM+ over an extended period of time.

**Table 2:** **Significance of differences in values presented in Table**1

**Variable**^***a***^	**Image stacks (#)**^***b***^	**Total biovolume (μm**^**3**^**/μm**^**2**^**)**^***b***^	**Mean thickness (μm)**^***b***^	**Roughness coefficient**^***b***^	**Total surface area × 10**^**7**^**(μm**^**2**^**)**^***b***^	**Surface to volume ratio (μm**^**2**^**/μm**^**3**^**)**^***b***^
**Time (under 20%EO**_**2**_**)**						
3d vs. 2d	10	Increase^*c*^ 0.0002	Increase <0.0001	Decrease <0.0001	NS^*d*^	Decrease 0.0027
6d vs. 2d	10	Increase 0.0002	Increase 0.0002	Decrease <0.0001	Decrease <0.0001	Decrease <0.0001
**Mucin concentration (3 d under 20%EO**_**2**_**)**						
2.0X vs. 1X	10	Increase 0.0002	Increase 0.0003	Decrease 0.0006	NS	Decrease 0.0018
0.5X vs. 1X	10	Increase 0.0019	Increase 0.0007	Decrease 0.0011	Increase 0.0290	NS
**DNA concentration (3 d under 20%EO**_**2**_**)**						
1.5X vs. 1X	10	Decrease <0.0001	Decrease <0.0001	Increase <0.0001	Decrease <0.0001	Increase <0.0001
0.5X vs. 1X	10	Decrease <0.0001	Decrease 0.0002	Increase 0.0013	Decrease 0.0008	Increase 0.0124
**Oxygen concentration (EO**_**2**_**)**^***e***^						
10% vs. 20%	10	Increase <0.0001	Increase <0.0001	Decrease <0.0001	Decrease <0.0001	Decrease <0.0001
0% vs. 20%	10	Decrease <0.0001	Decrease <0.0001	Increase <0.0001	Decrease 0.0287	Increase 0.0482

^*a*^All strains carry pMRP9-1 and were grown without shaking.

^*b*^See Table 1 for description of parameters.

^*c*^Significant change with *P* value indicated below direction of change.

^*d*^NS, no significant difference.

^*e*^20%, aerobic; 10%, microaerobic; 0%, anaerobic; cultures were grown for 3 d, except 0% EO_2_ for 6d.

### Mucin and DNA concentrations influence the development of the PAO1 BLS

**Figure 4:**
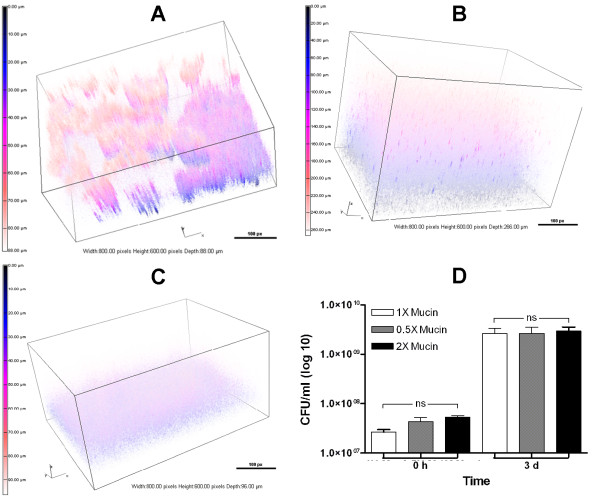
**Changing the level of mucin within ASM+ influences the development of PAO1 BLS.** Bacteria were inoculated in ASM+ containing 5 mg/ml (1X), 2.5 mg/ml (0.5X), or 10 mg/ml (2X) mucin and incubated for 3 d under 20% EO_2_/static conditions. The structures were analyzed by CLSM and 3-D images were constructed. Architecture of PAO1 BLS formed in the presence of 1X (**A**), 0.5X (**B**), or 2X (**C**) mucin. Boxes, 800.00 px W x 600.00 px H; bars, 100 px. (D) After 3 d, the gelatinous mass was removed from each well and vortexed to suspend the bacteria. The bacterial suspension was serially diluted and aliquots from each dilution were spotted on LB agar to determine the CFU/ml. Values represent the means of at least three independent experiments ± SEM.

Mucin, together with extracellular DNA, contributes to the viscosity of the CF sputum [17,18]. Mucin is one of the main components of ASM+. To determine if variations in the amount of mucin or DNA in ASM+ would affect the formation of the BLS, we adjusted the concentration of each component individually. With 0.5X mucin (2.5 mg/ml) or 2X mucin (10 mg/ml), PAO1 formed BLS, but the architecture was more diffuse in appearance than BLS seen with 1X mucin (5 mg/ml) (Figure 4). In general, varying the mucin concentration altered the structural parameters of PAO1 BLS. Either reduced or elevated mucin concentrations increased the biovolume and thickness significantly while the roughness was significantly decreased in both cases (Tables 1 and 2). Additionally, 0.5X mucin significantly increased the total surface area, while 2X mucin reduced the surface to biovolume ratio significantly (Table 2). We eliminated the possibility that variations in the amount of mucin simply affected the growth of PAO1 by determining CFU/ml for PAO1 grown in ASM+ with 1X, 0.5X or 2X mucin. After 3 d, comparable growth was observed in each condition, approximately 5 X 10^9^ CFU/ml (Figure 4D).

**Figure 5:**
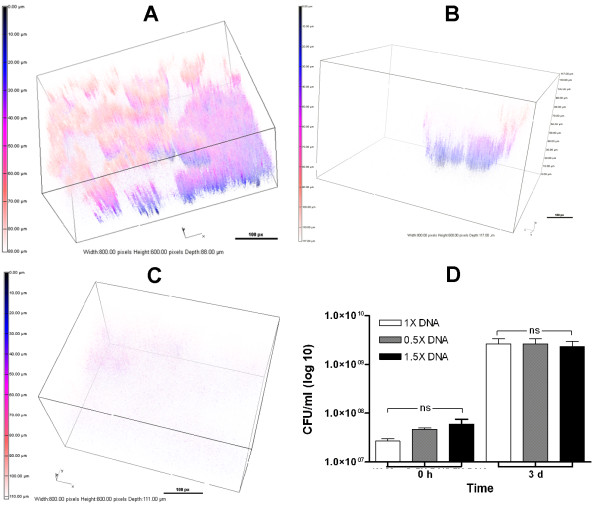
**Variations in the level of DNA within ASM+ affect the development of PAO1 BLS.** ASM+ containing 4 mg/ml (1X), 2 mg/ml (0.5X), or 6 mg/ml (1.5X) unsheared salmon sperm DNA was inoculated with PAO1/pMRP9-1 and incubated for 3 d under 20% EO_2_/static conditions. The structures were analyzed by CLSM and 3-D images were constructed. Architecture of PAO1 BLS formed in the presence of 1X (**A**), 0.5X (**B**), or 1.5X (**C**) DNA. Boxes, 800.00 px W x 600.00 px H; bars, 100 px. (**D**) After 3 d, the gelatinous mass was removed from each well and vortexed to suspend the bacteria. The bacterial suspension was serially diluted and aliquots from each dilution were spotted on LB agar to determine the CFU/ml. Values represent the means of at least three independent experiments ± SEM.

Variation in the amount of DNA produced more dramatic differences. When the amount of DNA was reduced to 0.5X (2 mg/ml), BLS were detected but confined to a small area of the gelatinous mass rather than spread throughout the medium as observed with 1X DNA (Figure 5A, B). When we increased the amount of DNA to 1.5X (6 mg/ml), individual cells were found scattered throughout the gelatinous medium, but no defined structures were detected (Figure 5C). The total biovolume, mean thickness, and total surface area of BLS developed in the presence of either 0.5X or 1.5X DNA were significantly less than those of BLS developed in the presence of 1X DNA (Tables 1 and 2). In contrast, the values of the roughness coefficient and surface to biovolume ratio were significantly increased (Table 2). This resembles the initial stage of biofilm development on an abiotic surface in which *P. aeruginosa* colonizes the surface and forms a single monolayer. As for the variations in mucin, we enumerated the CFU/ml for PAO1 grown in ASM+ with 1X, 0.5X or 1.5X DNA, and again, comparable levels of growth were obtained in each condition (Figure 5D).

### The level of EO_2_ affects the formation of BLS

**Figure 6:**
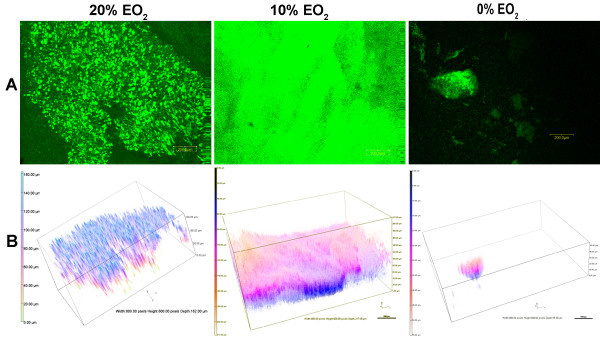
**The level of EO**_**2**_**influences the development of PAO1 BLS in ASM+.** Cells were inoculated into ASM+ and the cultures were incubated for 3 d under 20% or 10% EO_2_. To obtain growth of PAO1 anaerobically, 10% potassium nitrate was added as a terminal electron acceptor and incubation continued for 6 d in 0% EO_2_. The biofilms were analyzed as described in Figure 3. (**A**) CLSM micrographs of the BLS; magnification, 10X; bar, 200.00 nm. (**B**) The 3-D architecture of the BLS shown in (A); boxes, 800.00 px W x 600 px H; maximum depth, 20% EO_2_ 88.00 μm, 10% EO_2_ 217.00 μm, 0% EO_2_ 56.00 μm; bar, 100 px.

Previous studies suggested that within the lung alveoli of CF patients, *P. aeruginosa* survives and grows under an oxygen gradient of 10% EO_2_ to 0% EO_2_[5,21,22]. The experiments described above were conducted under 20% EO_2_. Therefore, we compared the development of the PAO1/pMRP9-1 BLS in ASM+ under 20%, 10% and 0% EO_2_. Cultures were incubated for 3 d under 20% and 10% EO_2_. For growth under 0% EO_2_, ASM+ was supplemented with 10% potassium nitrate as a terminal electron acceptor [5] and incubated for 6 d. Under anaerobic conditions, *P. aeruginosa* grows rapidly using anaerobic respiration, which requires nitrate (NO_3_^−^), nitrite (NO_2_^−^), or nitrous oxide (N_2_O) as alternative terminal electron acceptors [5]. As *P. aeruginosa* penetrate the thick mucus within the lung alveoli of CF patients and reach the hypoxic zone, they transit from aerobic to anaerobic metabolism and begin to utilize the NO_3_^−^ and or NO_2_^−^ present within the CF mucus [5]. Compared with structures that formed under 20% EO_2_, those that formed under 10% EO_2_ appeared more developed by CLSM (Figure 6A), much more dense and reaching almost twice the maximum depth (Figure 6B). Quantitative structural analysis by COMSTAT confirmed that compared with 20% EO_2_, the growth of PAO1 under 10% EO_2_ significantly increased the biovolume and mean thickness of the BLS (Tables 1 and 2). However, the values for the roughness coefficient, surface area, and surface to biovolume ratio were significantly reduced (Tables 1 and 2). In contrast, structures developed under 0% EO_2_ were smaller and limited to only a small portion of the gelatinous mass within the well (Figure 6). These structures were much less developed than BLS formed under 20% EO_2_ as shown by the significantly reduced mean thickness, total biovolume, and surface area (Tables 1 and 2). However, the roughness coefficient and surface to biovolume were significantly increased (Tables 1 and 2). These results suggest that in ASM+, maximum development of the PAO1 BLS occurs under 10% EO_2_, whereas the growth under 0% EO_2_ severely limits their development. Based on this finding, we conducted the rest of the PAO1 BLS analysis under 10% EO_2_.

### Different *P. aeruginosa* strains produce dissimilar BLS in ASM+

**Figure 7:**
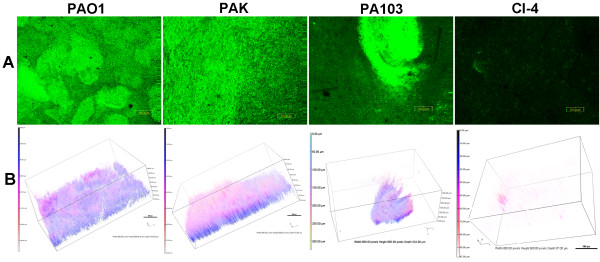
***P. aeruginosa *strains vary in their ability to develop BLS in ASM+.***P. aeruginosa* strains PAK, PA103, and CI-4 (a clinical isolate) were transformed with pMRP9-1. The strains, plus PAO1/pMRP9-1, were grown in ASM+ under 10% EO_2_ without shaking for 3 d. The BLS were analyzed as described in Figure 3. (**A**) CLSM micrographs of the BLS; magnification, 10X; bar, 200.00 nm. (**B**) The 3-D architecture of the BLS shown in (A); boxes, 800.00 px W x 600 px H; bars, 100 px.

As there are many strains of *P. aeruginosa* that differ in their ability to produce conventional biofilm, we compared the development of the BLS by PAK and PA103 under 10% EO_2_ with that of PAO1. These strains were originally isolated from infected patients and have been extensively utilized in *in vitro* and *in vivo* virulence studies [10,23-26]. Additionally, we examined the *P. aeruginosa* strain CI-4, a clinical isolate obtained from a patient with a chronic lower respiratory infection (30 days with the same strain) [27]. These strains were transformed with pMRP9-1 (for GFP expression) and grown in ASM+ for 3 d and the BLS analyzed as described in Methods. None of the PAK BLS parameters were significantly different from those of PAO1 BLS (Tables 3 and 4; Figure 7). With respect to PA103 BLS, only the total biovolume and mean thickness were significantly reduced in comparison with PAO1 BLS (Table 3 and 4; Figure 7). In contrast, CI-4 produced BLS that were significantly lower than those of PAO1 BLS in total biovolume, mean thickness, and total surface area but significantly higher than PAO1 in roughness coefficient and surface to biovolume ratio, indicating dispersal of poorly formed BLS throughout the gelatinous mass (Tables 3 and 4; Figure 7). These results indicate that *P. aeruginosa* strains differ in their ability to produce BLS within the ASM+.

**Table 3:** **Structural analysis of BLS formed by *P. aeruginosa *strains and QS mutants**

**Strains**^***a***^	**Image stacks (#)**^***b***^	**Total biovolume (μm**^**3**^**/μm**^**2**^**)**^***b***^	**Mean thickness (μm)**^***b***^	**Roughness coefficient**^***d***^	**Total surface area × 10**^**7**^**(μm**^**2**^**)**^***b***^	**Surface to volume ratio (μm**^**2**^**/μm**^**3**^**)**^***b***^
**Prototrophs and clinical isolate**						
PAO1	10	18.2 ± 0.69	17.5 ± 0.12	0.05 ± 0.01	0.73 ± 0.23	0.28 ± 0.07
PAK	10	13.7 ± 2.82	13.2 ± 2.62	0.05 ± 0.02	0.62 ± 0.05	0.27 ± 0.06
PA103	10	10.7 ± 0.08	12.6 ± 2.13	0.07 ± 0.03	1.32 ± 0.50	0.61 ± 0.21
CI-4	10	0.48 ± 0.17	0.77 ± 0.45	1.67 ± 0.12	0.23 ± 0.84	2.45 ± 0.02
**Quorum-sensing mutants**						
PAO1 (wt)	10	18.2 ± 0.69	17.5 ± 0.12	0.05 ± 0.01	0.73 ± 0.23	0.21 ± 0.07
PAO-R1 (Δ*lasR*)	10	19.3 ± 0.43	18.0 ± 0.00	0.02 ± 0.00	0.43 ± 0.15	0.12 ± 0.04
PAO-JP1 (Δ*lasI*)	10	17.6 ± 1.45	17.8 ± 0.15	0.02 ± 0.02	0.65 ± 0.26	0.22 ± 0.11
PDO111 (Δ*rhlR*)	10	7.29 ± 0.10	8.26 ± 0.05	0.13 ± 0.01	1.10 ± 0.08	0.79 ± 0.04
PDO100 (Δ*rhlI*)	10	6.61 ± 2.25	8.65 ± 2.49	0.67 ± 0.12	0.98 ± 0.14	1.01 ± 0.23
PW2798^*c*^ (Δ*pqsA*)	10	18.4 ± 0.30	17.7 ± 0.08	0.03 ± 0.01	0.70 ± 0.10	0.20 ± 0.03

^*a*^All strains carry pMRP9-1 and were grown for 3 d under 10% EO_2_ without shaking.

^*b*^See Table 1 for description of parameters.

^*c*^PW2798::*pqsA-lac.*

**Table 4:** **Significance of differences in values presented in Table**3

**Variable**^***a***^	**Image stacks (#)**^***b***^	**Total biovolume (μm**^**3**^**/μm**^**2**^**)**^***b***^	**Mean thickness (μm)**^***b***^	**Roughness coefficient**^***b***^	**Total surface area × 10**^**7**^**(μm**^**2**^**)**^***b***^	**Surface to volume ratio** **(μm**^**2**^**/μm**^**3**^**)**^***b***^
**Prototrophs and clinical isolate**						
PAK vs. PAO1	10	NS^*c*^	NS	NS	NS	NS
PA103 vs. PAO1	10	Decrease^*d*^ 0.0004	Decrease 0.0313	NS	NS	NS
CI-4 vs. PAO1	10	Decrease <0.0001	Decrease <0.0001	Increase <0.0001	Decrease 0.0417	Increase <0.0001
**Quorum-sensing mutants**						
PAO-R1 vs. PAO1	10	NS	Increase 0.0241	Decrease 0.0172	NS	NS
PAO-JP1 vs. PAO1	10	NS	NS	NS	NS	NS
PDO111 vs. PAO1	10	Decrease <0.0001	Decrease <0.0001	Increase 0.0027	NS	Increase <0.0001
PDO100 vs. PAO1	10	Decrease 0.0026	Decrease 0.0120	Increase 0.0020	NS	Increase 0.0175
PW2798 vs. PAO1	10	NS	NS	NS	NS	NS

^*a*^All strains carry pMRP9-1 and were grown under 10% EO_2_ without shaking.

^*b*^See Table 1 for description of parameters.

^*c*^NS, no significant difference.

^*d*^Significant change with *P* value indicated below direction of change.

### Quorum sensing affects the development of PAO1 BLS in ASM+

The three quorum sensing (QS) systems *las*, *rhl*, and *pqs* contribute to the development of *P. aeruginosa* biofilms [28-30]. Mutants defective in one or more of these systems failed to form well developed biofilms compared with the PAO1 parent strain [28-30]. Using a conventional biofilm medium (LB broth), we compared the biofilm developed on a plastic cover slip in a microtiter plate well by PAO1 and its *lasR, lasI, rhlR, rhlI,* and *pqsA* mutants. With the exception of the medium, the biofilms were developed under the same conditions that we used to develop the BLS. Compared with PAO1, all QS mutants produced reduced biofilms (data not shown). We then examined the contribution of the QS systems to the formation of the PAO1 BLS in ASM+, by comparing the structures formed by PAO1 with those formed by these same QS mutants. The mutants were transformed with pMRP9-1 and the development of the BLS by the transformants was examined under 10% EO_2_ for 3 d at 37°C.

**Figure 8:**
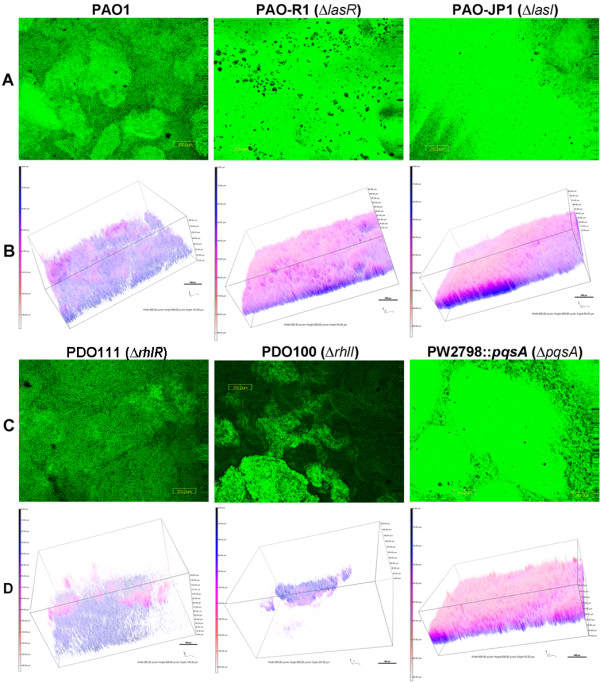
**Loss of individual QS genes affects BLS formation.** PAO1 strains defective in the *lasR* (PAO-R1), *lasI* (PAO-JP1), *rhlR* (PDO111), *rhlI* (PDO100), or *pqsA* (PW2798::*pqsA-lacZ*) genes were transformed with pMRP9-1 and the transformants plus PAO1/pMRP9-1 as a control were grown in ASM+ under 10% EO_2_ without shaking for 3 d. The BLS were analyzed as described in Figure 3. (**A** and **C**) Representative micrographs of the BLS; magnification, 10X; bar, 200.00 nm. (**B** and **D**) Respective 3-D images constructed from the CLSM micrographs. Boxes, 800.00 px W x 600 px H; bars, 100 px.

The *las* mutants PAO-R1 (Δ*lasR*) and PAO-JP1 (Δ*lasI*) produced BLS that visually and architecturally resembled each other (Figure 8A, B). With respect to the five tested parameters, BLS produced by PAO-JP1 were not significantly different from those BLS produced by PAO1 (Tables 3 and 4). The mean thickness of BLS produced by PAO-R1 was significantly higher than that of PAO1 BLS while the roughness coefficient was significantly lower (Tables 3 and 4). The *pqs* mutant PW728::*pqsA-lacZ* produced BLS that were not significantly different from PAO1 BLS (Figure 8; Tables 3 and 4). The biovolume and mean thickness of BLS produced by either the *rhlI* mutant (PDO100) or *rhlR* (PDO111) were significantly less than those produced by PAO1 (Figure 8; Tables 3 and 4). In contrast the values of the roughness coefficient and the surface to biovolume ratio were significantly higher than those for PAO1 BLS (Figure 8; Table 3 and 4). These results suggest that among all three QS systems, *rhlI* and *rhlR* have a major impact on the development of BLS in ASM+ by PAO1.

### *S. aureus* develops BLS under 20**%** EO_2_ but not 10**%** EO_2_

**Figure 9:**
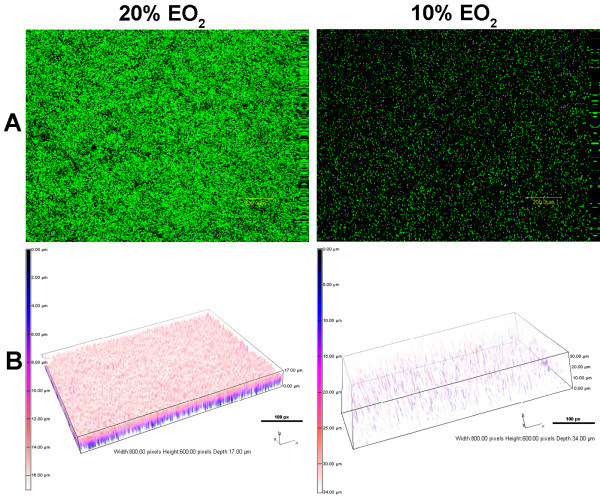
**Growth under 10**% **EO**_**2**_**reduces *S. aureus *AH133 BLS development.***S. aureus* strain AH133 was grown in ASM+ under 20% EO_2_ or 10% EO_2_ without shaking for 3 d. The BLS were analyzed as described in Figure 3. (**A**) Representative micrographs of the BLS; magnification, 10X; bar, 200.00 nm. (**B**) Respective 3-D images constructed from the CLSM micrographs. Boxes, 800.00 px W x 600 px H; bars, 100 px.

*S. aureus* is one of the first microorganisms that colonize and grow within the thick mucus in the lung alveoli of CF patients [8]. Thus, we determined whether *S. aureus* would develop BLS in ASM+ under 20% or 10% EO_2_. The *S. aureus* strain AH133 which carries the GFP plasmid pCM11, was grown for 3 d at 37°C. Under 20% EO_2_, AH133 produced a well developed BLS within the entire gelatinous mass (Figure 9). However, under 10% EO_2_, the structures were far less developed with individual cells/small microcolonies scattered within the gelatinous mass (Figure 9). Compared to BLS produced under 20% EO_2_, total biovolume, mean thickness, and surface area of BLS produced under 10% EO_2_ were significantly reduced (*P* < 0.0001 for each value) (Table 5). In contrast, the roughness coefficient and surface to biovolume ratio values were significantly increased (*P* < 0.0001 for each value) (Table 5). This suggests that unlike *P. aeruginosa*, *S. aureus* produces more developed BLS under 20% EO_2_ rather than under 10% EO_2_.

**Table 5:** **Effect of oxygen on *Staphylococcus aureus *AH133 BLS**^***a***^

**EO**_**2**_	**Image stacks (#)**^***b***^	**Total biovolume (μm**^**3**^**/μm**^**2**^**)**^***b***^	**Mean thickness (μm)**^***b***^	**Roughness coefficient**^***b***^	**Total surface area × 10**^**7**^**(μm**^**2**^**)**^***b***^	**Surface to volume ratio** **(μm**^**2**^**/μm**^**3**^**)**^***b***^
20%	9	7.00 ± 0.46	7.57 ± 0.50	0.58 ± 0.17	0.76 ± 0.12	0.57 ± 0.09
10%	9	0.22 ± 0.03	0.27 ± 0.04	1.90 ± 0.02	0.07 ± 0.00	1.59 ± 0.01

^*a*^Strains were grown for 3 d without shaking.

^*b*^See Table 1 for description of parameters.

### *P. aeruginosa* eliminates BLS established by *S. aureus* within ASM+

**Figure 10:**
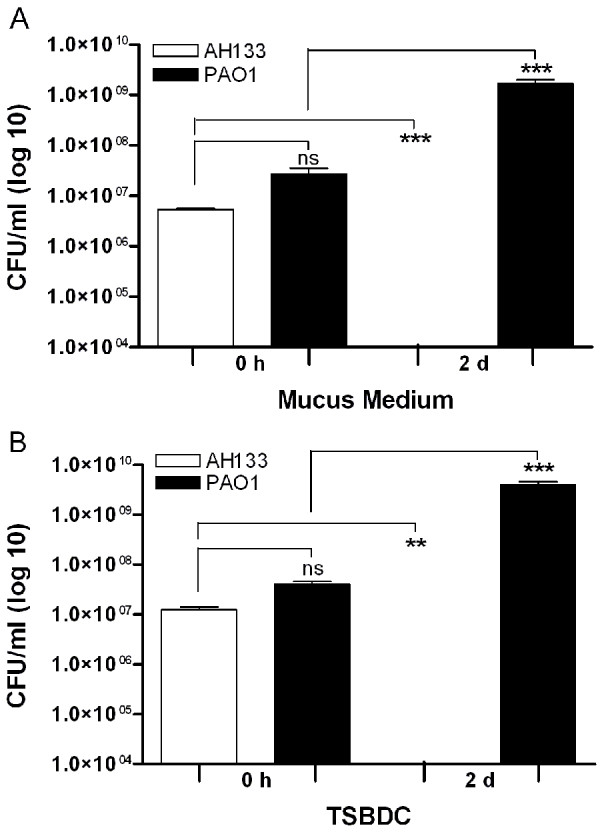
**PAO1 inhibits AH133 in co-culture.** Overnight LB cultures of AH133 and PAO1/pMRP9-1 were pelleted, washed, and resuspended in PBS. Resuspended cells of each species were inoculated into ASM+ (**A**) or TSBDC (**B**) at an initial OD_600_ of approximately 0.015. The CFU/ml of each species was determined at the time of inoculation (0-time) and after 48 h of growth using selective media (Methods). The graphs show CFU/ml obtained from BLS in ASM+ (**A**) and planktonic growth in TSBDC (**B**). Values represent the means of at least three independent experiments ± SEM.

The lungs of CF patients are colonized with a variety of pathogens, including *S. aureus*, *P. aeruginosa*, and *K. pneumoniae*, over the course of time [1]. However, as the disease progresses, the predominate pathogen within the CF infected lung is *P. aeruginosa*[1,8]. Previous studies showed that QS-controlled extracellular factors produced by *P. aeruginosa*, including quinoline molecules and LasA, inhibited the planktonic growth of *S. aureus* and *S. epidermidis*[31,32]. Additionally, recent studies showed that the *P. aeruginosa* extracellular polysaccharide as well as the organic compound *cis*-2-decenoic acid disrupted established biofilms produced by Gram-positive bacteria [33]. Therefore, we first determined if PAO1 inhibits the growth of the *S. aureus* strain AH133 in ASM+. We co-inoculated ASM+ with approximately 1 x 10^7^ CFU/ml each of PAO1 and AH133 and incubated the culture for 48 h at 37°C under 20% EO_2_. After 48 h, about 1 x 10^9^ CFU/ml of PAO1 was recovered from the gelatinous ASM+ mass, but no CFU of AH133 (Figure 10A). We obtained similar results when we repeated the experiments using TSBDC, a growth medium that supports planktonic growth of both organisms (Figure 10B). These results suggest that similar to other previous observations, *P. aeruginosa* eliminates *S. aureus*, when the two are grown together at the same time.

**Figure 11:**
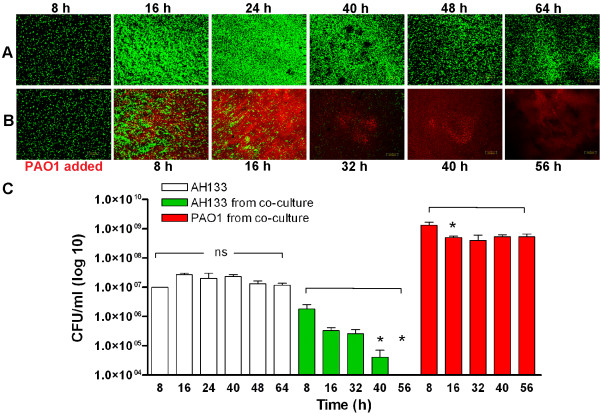
**Elimination of AH133 BLS is due to the bactericidal effect of PAO1.** PAO1/p7605 (red) gradually eliminates previously-formed AH133 (green) BLS. ASM+ was inoculated with AH133 and the cultures were grown for 8 h to allow for the partial development of AH133 BLS. (**A**) One culture was continued without addition of PAO1 for a total of 64 h. (**B**) The other culture was inoculated with PAO1/p7605 (starting density similar to that used to initiate the AH133 culture). Incubation was continued for an additional 56 h and cultures were analyzed at 8- to 16-h intervals by CLSM (exact time points indicated above and below micrographs). (**C**) PAO1 is bactericidal to AH133. Two sets of wells containing ASM+ were inoculated with AH133 and incubated for 8 h. PAO1/pMP7605 was added to one set of wells and incubation of both sets was continued for 56 h. At the specified time points, the gelatinous mass was obtained and the CFU/ml of each species was determined using selective media (Methods). White bars: AH133 CFU/ml in single culture; green bars, CFU/ml of AH133 in the co-culture; red bars, CFU/ml of PAO1/pMP7605 from the co-culture. Values represent the means of at least three independent experiments ± SEM.

To simulate the scenario in which *S. aureus* colonizes the CF lung first and *P. aeruginosa* follows, we then examined the effect of PAO1 on partially developed (8 h) AH133 BLS. As AH133 expresses GFP, we transformed PAO1 with pMP7605, a plasmid from which RFP is expressed constitutively [34], to allow visualization of each strain within the BLS. Individually, the strains produced well developed BLS in ASM+ (Figures 2, 10A). At 8 h post inoculation, AH133 formed a defined structure (Figure 11A). We then added PAO1/pMP7605 (at a starting density similar to that used to initiate the AH133 culture) and continued incubation for 56 h. The cultures were analyzed at 8- and 16-h intervals to 64 h for the AH133 alone and 56 h post addition of PAO1/pMP7605 for changes in the BLS produced by AH133 and the development of any PAO1 BLS (Figure 11A, B). At 16 and 24 h post-initiation, AH133 produced well-developed mature BLS (Figure 11A). The AH133 BLS changed in appearance over the rest of the time course, but did not disappear (Figure 11A). In contrast, in the dual culture, the AH133 structure was considerably reduced at the corresponding time points (Figure 11B). By 32 h only remnants of the AH133 BLS remained, and by 40–56 h, the AH133 BLS appeared to be completely replaced by well-developed PAO1 BLS (Figure 11B). The regression of AH133 structure appears to be due to the expansion of the PAO1 structure at 8, 16, and 32 h post-initiation (Figure 11B).

This observed phenomenon could be due to the dispersion of the AH133 BLS or a bactericidal effect of PAO1 on AH133. Therefore, at each time point, the gelatinous masses containing AH133 alone or AH133 plus PAO1 were vortexed, serially diluted, and the CFU/ml determined. Aliquots of each dilution were spotted on *Pseudomonas* isolation agar for *P. aeruginosa* and mannitol salt agar for *S. aureus*. At all tested time points, the CFU/ml of the single AH133 biofilm was similar (about 1 x 10^7^) (Figure 11C, white bars). However, the CFU/ml of AH133 within the mixed BLS was visibly reduced 8 h after addition of PAO1 and significantly reduced at 40 and 56 h, with no CFU of AH133 recovered 56 h post addition of PAO1 (Figure 11C, green bars). In contrast, the CFU/ml of PAO1/pMP7605 within the mixed BLS dropped between 8 and 16 h post biofilm initiation but did not change significantly after 16 h (Figure 11C, red bars). These results suggest that PAO1 exerts a bactericidal effect, and that the development of the *P. aeruginosa* BLS in the co-culture proceeded at the expense of the *S. aureus* BLS.

## Discussion

CF sputum is a highly viscous secretion in which PAO1 grows readily. PAO1 forms conventional biofilms on abiotic surfaces [13,19,35], but it develops macrocolonies, tight aggregates consisting of numerous microcolonies, in ASM and the CF lung [16,21]. While PAO1 formed a typical flat undifferentiated biofilm that completely covered the substratum with a homogenous distribution of the biovolume in a continuous flow-through system, it grew almost exclusively as discrete microcolonies that eventually formed a mature biofilm on a mucin-covered glass surface [19]. Based on these results, Landry *et al*. suggested that mucin interacts with specific PAO1 adhesins thereby immobilizing the bacteria onto the glass surface [19]. In our analysis, the observed BLS developed exclusively within the gelatinous mass formed by ASM+ and not on the surface of the well (Figure 1). It is likely that through the initial interaction of these putative adhesins, individual PAO1 bacteria adhere to the mucin glycoprotein forming the nuclei of the microcolonies and leaving no bacteria to adhere to the plastic surface. Individual nuclei would then recruit more PAO1 bacteria, possibly through additional inter-bacterial and glycoprotein-bacterial interactions, growing into well-defined BLS observed over time (Figure 2).

Based on the work of Ghani & Soothill [15] and Sriramulu *et al*. [16], we utilized 0.5% mucin (1X) in our ASM+. But more recently, Henke *et al*. [36], showed that the concentrations of MUC5AC and MUC5B, the principal gel-forming mucins, are decreased in airway secretions from CF patients with stable disease and greatly increased during pulmonary exacerbations (by 89% and 908%, respectively). When we reduced the mucin concentration of ASM+ by 50% (0.5X), the gelatinous mass still formed in the well, possibly through the contribution of other ASM+ components (DNA and lecithin) that add to the viscosity. However, the typical multilayered BLS was eliminated and replaced with a structure that appears to consist of small microcolonies amid individual cells and tiny cell clusters distributed throughout most of the gelatinous mass (Figure 4A, B). Surprisingly, the effect of increasing the concentration of mucin to 2X on the development of BLS was similar to that induced by reducing the mucin concentration. Rather than the distinct highly structured BLS architecture, PAO1 produced small microcolonies distributed throughout the ASM+ (Figure 4C). At this time, we do not know if the increase in the availability of mucin glycoprotein interferes with the development of microcolonies that coalesce to form the well-developed BLS.

One of the hallmarks of the CF syndrome is the overproduction of mucin within the lung alveoli [1,3,7]. Yet during *P. aeruginosa* infection of the CF lung alveoli, the level of mucin may vary [36]. *P. aeruginosa* LPS induces the production of reactive oxygen intermediates, which cause release of transforming growth factor α; TGF-α then up-regulates the expression of MUC-5 AC thereby causing excessive mucin production [37-39]. However, *P. aeruginosa* produces other factors that may reduce the amount of mucus within its immediate vicinity; alveolar mucin is degraded by *P. aeruginosa* extracellular serine proteases such as LasB [40]. Ultimately, the interaction of all these factors would produce a net mucin concentration suitable for the full development of the BLS, while any imbalance in the production of these factors that reduces or increases mucin concentration would prevent the establishment of the BLS. Alternatively, BLS may form in the initial stages of *P. aeruginosa* infection in the CF lung. Treatment that reduces the amount of mucin present would disperse the bacteria making them more susceptible to antibacterial treatment (stable disease). Alternatively, mucin may reduce the chances of forming new BLS.

Extracellular DNA is another contributor to the viscosity of CF sputum [15,16]. Within the CF lung, there are several sources for this extracellular DNA – dead host immune cells, lysed bacteria, QS-regulated release of *P. aeruginosa* DNA, and outer membrane vesicles that contain DNA [41,42]. Like mucin, DNA contributes to biofilm formation as well; a similar biofilm was detected on a glass surface coated with DNA [41]. Previous studies using other biofilm development media, such as LB or minimal medium, indicated that extracellular DNA is critical for the initial establishment of *P. aeruginosa* biofilms [42]. The levels of extracellular DNA also vary within CF sputum, ranging from 0.3 to 9.5 mg/ml in one study of 167 CF sputum samples [43]. Variations in the level of extracellular DNA in ASM+ affected the development of BLS much more dramatically than variations in the level of mucin. In ASM+ with 0.5X DNA (2 mg/ml), a well developed BLS was visible (Figure 5B), but the biovolume and total surface area occupied were considerably less (Table 1 and 2). When the amount of DNA was increased to 1.5X (6 mg/ml), PAO1 did not produce detectable structures; rather, the gelatinous mass formed by the ASM+ contained scattered individual cells (Figure 4C). However, at this time it is not clear how an increase in the external DNA reduces the number of BLS within the gelatinous mass of ASM+.

Within the lung of CF patients and during other chronic lung infections, *P. aeruginosa* survives under microaerobic (10% EO_2_) to anaerobic (0% EO_2_) conditions. A steep oxygen gradient exists within the *P. aeruginosa* infected alveolar mucus [5,21]. Within the mucus, *P. aeruginosa* secretes compounds that lower the oxygen transfer rate generating optimum conditions for microaerobic growth [22,44]. We showed previously that lower oxygen tension also influences the expression of *P. aeruginosa* virulence genes [45]. Compared with aerobic conditions, the expression of pyoverdine genes was reduced under microaerobic conditions; in contrast, the expression of the exotoxin A gene, *toxA* was increased [45]. Compared with 20% EO_2_ and 0% EO_2_, microaerobic (10% EO_2_) conditions are optimal for the development of *P. aeruginosa* BLS in ASM+. BLS developed under 10% EO_2_ had a greater mean thickness and a larger biovolume than those developed under either 20% or 0% EO_2_ (Figure 6, Table 1 and 2). In the absence of EO_2_, PAO1 required 6 days to develop rudimentary BLS (Figure 6C) indicating that a low level of oxygen is essential for the full development of these structures.

Depending on conditions under which the biofilms were developed (medium, the biofilm development system, and the biofilm substrate), previous studies indicated the involvement of the QS systems in the development of *P. aeruginosa* biofilm [29,30,35,46]. In those studies, the deficiency in biofilm development was associated with either a *lasI* or *rhlI* mutation. We tested mutants defective in all three known *P. aeruginosa* QS systems in ASM+. PAO-R1 (Δ*lasR*), PAO-JP1 (Δ*lasI*), and PW2798::*pqsA-lacZ* (Δ*pqsA*) produced BLS that were visually and architecturally similar to each (Figure 8). In contrast, PDO111 (Δ*rhlR*) BLS were visually, architecturally, and structurally dissimilar to PAO1 BLS, in that they had a smaller biovolume and mean thickness (Figure 8, Tables 3 and 4). The loss of *rhlI* (PDO100) had the most profound effect on the BLS produced by PAO1 in ASM+. Both PDO100 (Δ*rhlI*) and PDO111 (Δ*rhlR)* produced BLS that were significantly smaller (biovolume, mean thickness) than PAO1 BLS (Figure 8, Tables 3 and 4). However, BLS produced by these two strains were more heterogeneous than PAO1 BLS (a significant increase in roughness coefficient) (Figure 8, Tables 3 and 4). Additionally, more regions of the PDO100 and PDO111 BLS were exposed to nutrients than PAO1 BLS (a significantly higher surface to biovolume values) (Figure 8, Tables 3 and 4). Our results suggest that the production and maturation of the fully-developed complex BLS requires a potential *P. aeruginosa* factor that is stringently controlled by the *rhl* and not the *las* or the *pqs* systems. Among the *P. aeruginosa* factors that are stringently controlled by the *rhl* system are the rhamnolipid biosurfactants [47,48]. The rhamnolipids encoded by the *rhlAB* operon contribute to biofilm development in *P. aeruginosa* through multiple mechanisms including maintaining open channels by affecting cell-to-cell interaction [28], promoting microcolony formation in the initial stages of biofilm development [49], and dispersing cells from the mature biofilms [50]. Analysis of PAOΔ*rhlA* and/or PAOΔ*rhlB* mutants in ASM+ should allow us to determine if rhamnolipid plays a role in the development of the BLS. Interestingly, PA103, which is known to have a deletion in *lasR*[51], produced BLS with reduced biovolume and mean thickness (compared with those produced by PAO1 or PAO-R1) (Figure 7, Tables 3 and 4). This suggests that the observed differences between the BLS produced by PAO1 and PA103 are not due to the loss of the *lasR* gene in PA103. CI-4, a clinical isolate obtained from a patient who had been continuously infected with *P. aeruginosa* for 30 days, has deletions in both *lasR* and *rhlR*[27]. This strain produced BLS that had less biovolume, mean thickness and covered less total surface area that PAO1; visually, the BLS were also unique in appearance among all the QS mutants, numerous small microcolonies distributed throughout the medium (Figure 7, Tables 3 and 4). This suggests that there is a complex interaction among the QS systems in controlling BLS production within ASM+.

Using ASM+, which has the same components as our ASM+, Sriramula *et al*. [16] examined the growth of PAO1, PAOΔ*lasR*, and PAOΔ*rhlR*. Both PAO1 and PAOΔ*rhlR* formed macroscopically visible clumps or aggregates, which they termed tight microcolonies, that could not be disturbed even with vigorous pipetting [16]. In contrast, PAOΔ*lasR* failed to develop these tight microcolonies [16]. In our study, neither PAO1, nor any other tested strain produced macroscopically visible structures. In part, this is due to the turbidity of ASM+. Similar to the tight microcolonies described by Sriramula *et al*. [16], the BLS we observed in our ASM+ did not attach to a surface. The BLS are adherent when fully-developed, but cells within the BLS can be dispersed by vortexing. Differences between the two studies, despite the similarity in the growth medium, are most likely due to differences in other growth conditions. While Sriramula *et al*. [16], grew their cultures under 20% EO_2_ with shaking, we grew our cultures under static conditions regardless of the EO_2_ concentration. Given these differences, it is not practical to directly compare the bacterial structures observed in the two studies with respect to the role of the QS systems in their formation.

Biofilms at different infection sites often consist of multiple species of bacterial pathogens [52,53]. These bacterial species may either compete with each other or support each other’s growth. Qin *et al*. [54] previously showed that *P. aeruginosa* inhibited the planktonic growth of *Staphylococcus epidermidis* through a QS-related mechanism. Additionally, using the static chamber cultivation system (microtiter plate assay), they demonstrated that *P. aeruginosa* extracellular polysaccharide disrupted an already established *S. epidermidis* biofilm [54]. Disruption of these biofilms, however, does not occur through the bactericidal effect observed with the planktonic cells; instead the bacteria within the biofilm were dispersed alive [54]. When we co-cultured *P. aeruginosa* and *S. aureus* statically under 20% EO_2_ in TSBDC or ASM+, *P. aeruginosa* eliminated *S. aureus* by day 2 (Figure 10). Furthermore, and similar to the findings by Qin *et al*. with *S. epidermidis*[54], the addition of *P. aeruginosa* to *S. aureus* BLS established in ASM+ disrupted the *S. aureus* BLS (11a, b). However, *P. aeruginosa* disrupted the *S. aureus* BLS through an bactericidal effect rather than dispersion. By 56-h post addition of PAO1, no CFU of AH133 were recovered (Figure 11C), although it is remotely possible that our failure to detect *S. aureus* is due to their existence in a viable but nonculturable state. This effect is similar to the clinical observations of CF lung infections where *S. aureus*, an early colonizer, is gradually replaced by *P. aeruginosa*. The nature of the PAO1 bactericidal factor that eliminates the *S. aureus* BLS is under investigation.

## Conclusions

In this study, we have demonstrated that thick, viscous ASM+ containing mucin and extracellular DNA and incubated under static conditions with lowered oxygen tension (10% EO_2_) – constituents and conditions similar to those within the lung alveoli of CF patients – induces the formation of biofilm-like structures by *P. aeruginosa* and *S. aureus*, two of the pathogens most commonly seen in the infected lungs of these patients. The BLS are not attached to the surface, but form within the medium as has been reported for the development of macrocolonies within the mucus in CF lungs. Thus, ASM+ represents an *in vitro* medium in which the effect of changing levels of substances produced by the host and the bacteria can be analyzed to determine the effect on such structures and on the susceptibility of the bacteria within the BLS to various treatments.

## Methods

### Bacterial strains, plasmids, and media

Bacterial strains and plasmids utilized in this study are described in Table 6. Strains were routinely grown in Luria Bertani (LB) broth under shaking conditions at 37°C. To analyze the development of biofilm-like structures, bacterial strains were grown in the previously described ASM+ [16]. To attain consistency from batch to batch of medium ASM+ was made in a quantity sufficient for each planned set of experiments, a stringent method of preparing the medium was used. The components were added into sterile water in exact order with vigorous vortexing for 10–30 seconds after each addition: mucin (Sigma-Aldrich, St. Louis, MO), 0.5% (w/v); unsheared salmon sperm DNA (Sigma-Aldrich), 0.4% (w/v); NaCl, 0.5% (w/v); KCl, 0.2% (w/v); casamino acids (Amresco, Solon, OH), 0.5% (w/v); egg yolk emulsion (source of lecithin; sterile; Remel, Lenexa, KS), 0.25% (v/v); diethylene triamine pentaacetic acid (1 mg/ml stock in 1 M NaOH; sterile; Sigma), 0.0005% (w/v). Finally, the pH was adjusted to 6.8. Antibiotics were then added to maintain sterility and for maintenance of plasmids: 300 μg carbenicillin/ml and/or 50 μg tetracycline/ml for *P. aeruginosa*; 10 μg erythromycin/ml for *S. aureus*. The completed medium was then vortexed for 2 minutes and again prior to pipetting. To induce biofilm formation on the substrate surface, we used trypticase soy broth dialysate (TSBDC) to which glycerol (1% v/v) and monosodium glutamate (0.5 M) were added [55].

**Table 6:** Strains and plasmids used in this study

**Strain**	**Description**	**Source**
**Plasmids**		
pCM11	Plasmid stable in *S. aureus* that constitutively expresses green fluorescent protein (GFP); Em^r^	Alexander Horswill, personal communication
pMRP9-1	pUCP-18 cloning vector carrying a GFP cassette; Cb^r^	[56]
pMP7605	pBBR1MCS-5 cloning vector carrying the mCherry gene under the control of the *tac* promoter; Cb^r^	[34]
***Pseudomonas aeruginosa***		
PA103	Human isolate	[24]
PAK	Prototroph; human isolate	[57]
PAO1	Prototroph; human isolate	[58]
PAO-R1	Δ*lasR* derivative of PAO1; Tc^r^	[51]
PAO-JP1	Δ*lasI* derivative of PAO1; Tc^r^	[59]
PDO111	*rhlR*::Tn*501* derivative of PAO1; Hg^r^	[60]
PDO100	Δ*rhlI*::Tn*501* derivative of PAO1; Hg^r^	[60]
PW2798::*pqsA-lacZ*	*pqsA-*H05::IS*lacZ*/hah derivative of PAO1; Tc^r^	[61]; University of Washington Genome Center
CI-4	Human isolate from chronic lower respiratory infection; Δ*lasR,* Δ*rhlR*	[27]
***Staphylococcus aureus***		
AH133	RN4220 carrying pCM11; Em^r^	[62]; Alexander Horswill, personal communication

Em, erythromycin; ^r^, resistant; Cb, carbenicillin; Hg, mercury; Tc, tetracycline.

To allow visualization of the bacteria, all *P. aeruginosa* strains were transformed by electroporation [63] with pMRP9-1 from which the gene for green fluorescent protein (GFP) is constitutively expressed [56]. To visualize PAO1 grown together with AH133, PAO1 was transformed with pMP7605 in which the mCherry gene that codes for red fluorescent protein (RFP) is expressed from the *tac* promoter [34]. The *S. aureus* strain AH133 carries the constitutive GFP plasmid pCM11 (AR Horswill, personal communication).

### Static microtiter plate culture system for development of the BLS

Bacteria were grown in a static microtiter plate culture system using sterile 24-well polystyrene plates (Falcon; BD, Franklin Lakes, NJ) [64,65]. Tested strains were grown overnight in LB broth. Cells were pelleted, washed, and resuspended in PBS. For analysis of the BLS formed by individual bacterial species, resuspended cells were inoculated in ASM+ to an initial OD_600_ of 0.02-0.03 and dispensed into the plate wells in 1 ml aliquots. For the analysis of BLS produced by two bacterial species, individual species were prepared and inoculated at an initial OD_600_ of 0.015. The plates were incubated at 37°C in static (nonshaking) conditions under environmental oxygen (EO_2_) concentration of 20**%** (aerobic), 10**%** (microaerobic), or 0**%** (anaerobic). Individual GasPak jars with Campy Pak Plus envelopes (BD) or GasPak EZ Anaerobic Pouches (BD) were used to generate the microaerobic and anaerobic EO_2_ conditions, respectively.

### Visualization of the BLS

This was done using confocal laser scanning microscopy (CLSM) [35,64]. The BLS were visualized within the wells of the microtiter plates using an Olympus IX71 Fluoview 300 confocal laser scanning microscope (Olympus America, Melville, NY). All images were obtained through a 203/0.40 Ph1 NA objective utilizing a green helium laser (546 nm) or argon laser (510–530 nm). Three-dimensional image reconstructions were performed using NIS-Elements 2.2 (Nikon Instruments, Melville, NY) to visualize the architecture of the BLS. All instrument settings were consistent for each individual experimental parameter tested.

### Quantitative structural analysis of the BLS

The number of image stacks obtained from the BLS was based on the greatest depth of the structures formed under the test conditions and was the same for all strains/conditions within an experiment (See Tables 1, 2, 3, 4). Each experiment was done in duplicate. Two 10-image stacks were obtained from random positions within each BLS (total 40-image stacks for each strain and/or condition). The 40-image stacks were analyzed using the COMSTAT program [20] for structural features of the BLS: biovolume, estimates the biomass of the BLS; mean thickness, a measure of spatial size of the BLS; roughness coefficient, a measure of how much the thickness of the BLS varies, or the heterogeneity of the BLS; total surface area, space occupied in each image stack; and surface to biovolume ratio, estimates the portion of the BLS exposed to nutrients (biovolume divided by the surface area of the substratum). Values represent the mean ± SEM.

### Quantification of the bacteria within the BLS

The highly viscous ASM+ forms a gelatinous mass in which the bacteria grow. Therefore, at the indicated time points, the mass from each well was transferred to a 1.5 ml microcentrifuge tube and vigorously vortexed to suspend the bacteria. The bacterial suspension was then serially diluted tenfold in PBS and 10 μl aliquots of each dilution were spotted on LB agar. For dual species experiments, the aliquots were spotted on *Pseudomonas* isolation agar (BD) to select for *P. aeruginosa* and mannitol salt agar (BD) to select for *S. aureus*. The plates were incubated at 37°C for 16 h and the colonies of microorganisms (CFU) were counted. The CFU/ml was determined using the following formula: CFU counted x dilution factor x 100.

### Statistical analyses

Statistical analyses of the results were done using GraphPad InStat 3.06 (GraphPad Software, San Diego, CA). One-way ANOVA with the Tukey-Kramer multiple comparisons post-test was used to determine significant differences over time and among treatments. The *t*-test was used to compare two strains or two treatments.

## Authors' contributions

CH designed portions of the study, conducted all experiments, and wrote the manuscript. ANH coordinated the project, designed portions of the study, and helped draft and revise the manuscript. JACH analyzed and interpreted data and critically revised the manuscript. All authors have read and approved the final manuscript.
